# Establishing baselines for echolocating bat activity at wind farms in mainland Southeast Asia

**DOI:** 10.1038/s41598-026-41384-8

**Published:** 2026-02-27

**Authors:** Neil M. Furey, Vuong Tan Tu, Alan Hitch, John Pilgrim, Mark Kunzer

**Affiliations:** 1 Harrison Institute, 15 St. Botolphs Road, Sevenoaks, TN13 3AQ UK; 2 Institute of Biology, Vietnam Academy of Science and Technology, No. 18, Hoang Quoc Viet Road, Nghia Do, Hanoi, Vietnam; 3 Department of Wildlife, Fish, and Conservation Biology, Museum of Wildlife and Fish Biology, University of California at Davis, Davis, CA 95616 USA; 4 School of Data Analytics and Computational Sciences, Harrisburg University of Science and Technology, Harrisburg, PA 17101 USA; 5 John Pilgrim Limited, The Old Post Office, The Street, Boyton, Woodbridge, Suffolk, IP123LW UK; 6 (Retired) Private Sector Investment Funds and Special Initiatives Division, Private Sector Operations Department, Asian Development Bank, 6 ADB Avenue, Mandaluyong, Metro Manila Philippines

**Keywords:** Acoustic monitoring, Bat fatalities, Southeast Asia, Wind energy, Climate sciences, Ecology, Ecology, Environmental sciences

## Abstract

Efforts to reduce bat fatalities caused by collisions with wind turbines in Southeast Asia are hampered by a lack of information on bat activity and factors influencing this at wind farms in the region. To address this deficit, we employed acoustic detectors to establish a baseline for bat activity at wind turbines in southern Vietnam for one year. Fieldwork confirmed the local occurrence of 22 bat species, whereas 11 were registered at the turbines. Bat activity at the turbines was greatest in May–October, although moderate-to-high and high levels of activity frequently occurred every month. Our findings suggest that rain and temperature may have limited utility for refining curtailment in the study area because they do not markedly reduce bat activity. However, this is unlikely to be true of the many areas experiencing greater rainfall and/or distinct winter periods in Southeast Asia. In demonstrating that increased windspeeds significantly reduce bat activity, our results also indicate curtailment at low wind speeds will be effective at reducing bat fatalities at wind farms in the region. As the first publicly available study to report bat activity and factors influencing this at a windfarm in Southeast Asia however, much remains to be learnt.

**Supplementary Information:**

The online version contains supplementary material available at 10.1038/s41598-026-41384-8.

## Introduction

Wind energy now accounts for > 10% of global power production and is one of the fastest-growing energy sectors worldwide due to the rapid push to decarbonise global power production^1^. Although widely considered a clean energy source, wind energy is not environmentally neutral. Wind farms are well known to negatively affect biodiversity as a result of habitat loss or degradation, displacement of wildlife and fatalities due to collisions^2,3^. Widespread and often extensive fatalities have highlighted the direct impacts of wind turbines on volant vertebrates^4,5^, such that these represent the leading cause of multiple mortality events for bats worldwide^6^. This presents a serious concern because bats reproduce slowly^7^ and collision fatalities at wind farms can cause rapid population declines, placing species at risk of local if not regional extinction^3,8^.

Southeast Asia currently has around 10.1 GW of operating wind power capacity at utility-scale, most of which is in Vietnam (≈ 6.5 GW), Thailand (≈ 2.1 GW), Philippines (≈ 0.7 GW), Laos (≈ 0.6 GW) and Indonesia (≈ 0.2 GW)^9,10^. This is set to increase dramatically in the near future because an additional ≈ 150 GW of prospective wind energy projects (i.e., projects announced or in pre-construction or construction stages) are documented for the region, although much of this is expected to occur offshore^10^. With over 385 bat species, Southeast Asia also supports a quarter of the world’s bat diversity, including many endemic taxa and the highest density of threatened bat species globally^11–14^. While there is no reason to expect lower fatality rates at wind turbines in the Old-World tropics than other regions, reviews have failed to find information on bat fatalities at windfarms in the region^2,15^. However, the very few projects that have released these to date^16^, coupled with undisclosed data we have seen from wind farms in several countries, demonstrates that significant mortalities can occur. As a result, efforts to address bat-turbine collisions in Southeast Asia are hampered by the paucity of publicly available information on patterns of bat activity, fatalities and the factors that influence these at wind farms across the region.

Studies have shown that environmental assessments undertaken before installation of wind turbines are poor predictors of subsequent fatality rates because the presence of turbines alters bat activity^17–19^. They have also shown that restricting blade rotation during periods of high collision risk is the most effective way to reduce bat fatalities currently available^3,20^. Since bat activity during turbine operation correlates strongly with fatalities^21^, quantifying bat activity with acoustic detectors as a function of time (i.e., nights, seasons) and weather conditions can provide powerful insights for predicting when bats are most at risk in poorly documented regions^2,22^. It can also help identify the best strategies for minimizing bat fatalities and avoiding power losses associated with blanket curtailment (i.e., the uniform stopping or reduction of blade rotation when night winds and temperatures cross specific thresholds, most often below windspeeds of 3.5–8 m/s and temperatures above 10 °C^23^.

In light of this, the purpose of our study was to (i) confirm the bat species routinely frequenting four turbine areas at a wind farm in southern Vietnam, (ii) quantify seasonal and nightly variation in bat activity at the turbines over a calendar year, (iii) evaluate the influence of weather and other factors on bat activity, and finally to (iv) determine if bats shift their activity to later in the night when wind speeds during dusk and early evening are high. Since Vietnam has ambitious targets for onshore and nearshore wind power and now plans to at least quadruple its current operating capacity of ≈ 6.5 GW to 26–38 GW by 2030^24^, this information has clear relevance for ensuring such increases can occur with minimal impacts on national bat populations.

## Results

### Bat species frequenting wind turbines

**Fig. 1:**
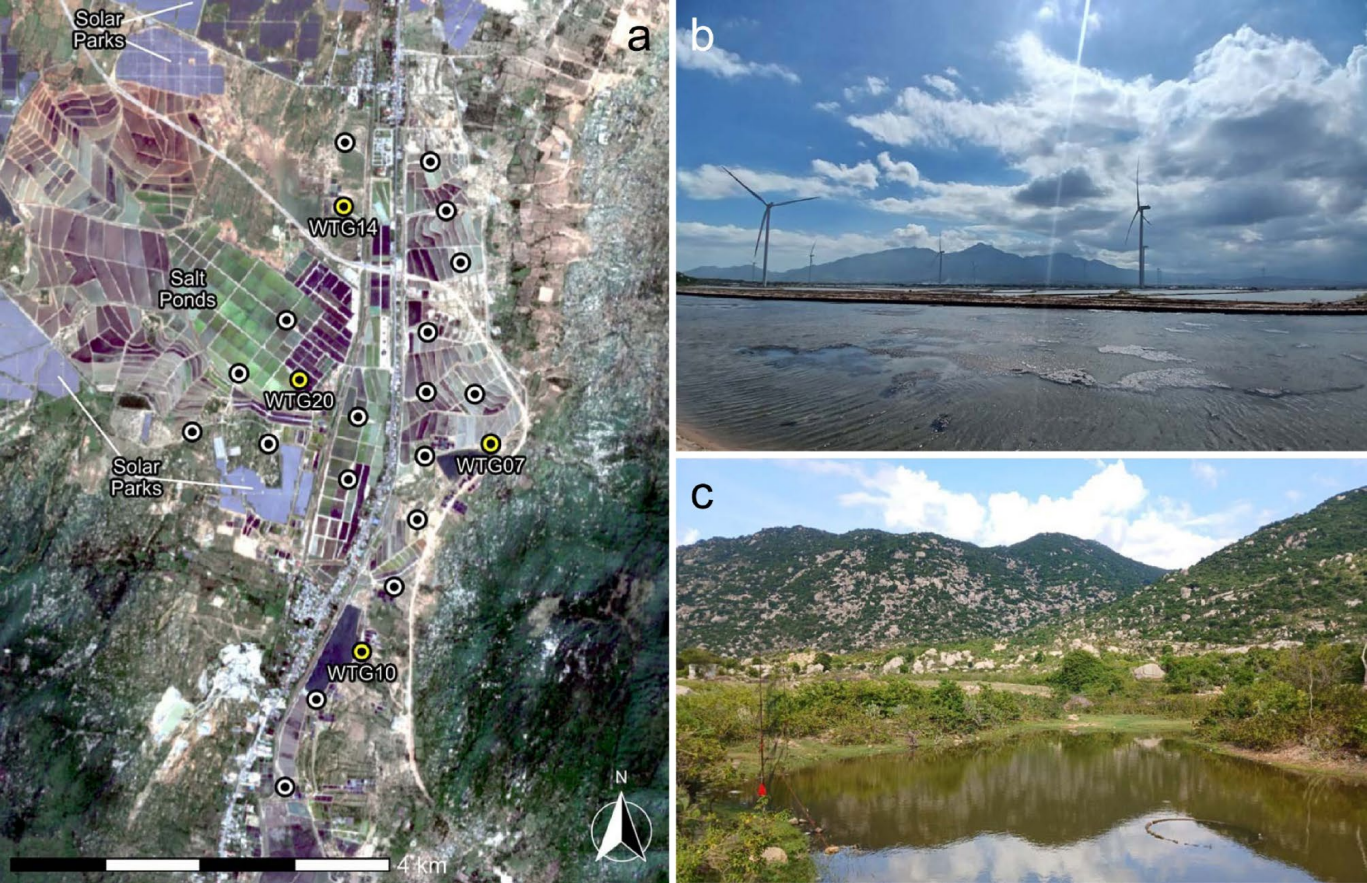
(**a**) Layout of wind turbines and four turbines monitored (WTG) in study area, (**b**) Salt ponds in central area of wind farm, with southwestern mountains in background, (**c**) Freshwater pond in foothills on eastern periphery of wind farm (next to WTG07). Satellite imagery were derived from Sentinel-2 MSI Level-2 A surface reflectance data, Copernicus Programme, European Space Agency. Processing and visualization were performed in R v4.5.2 within RStudio v2026.01.0.

**Fig. 2:**
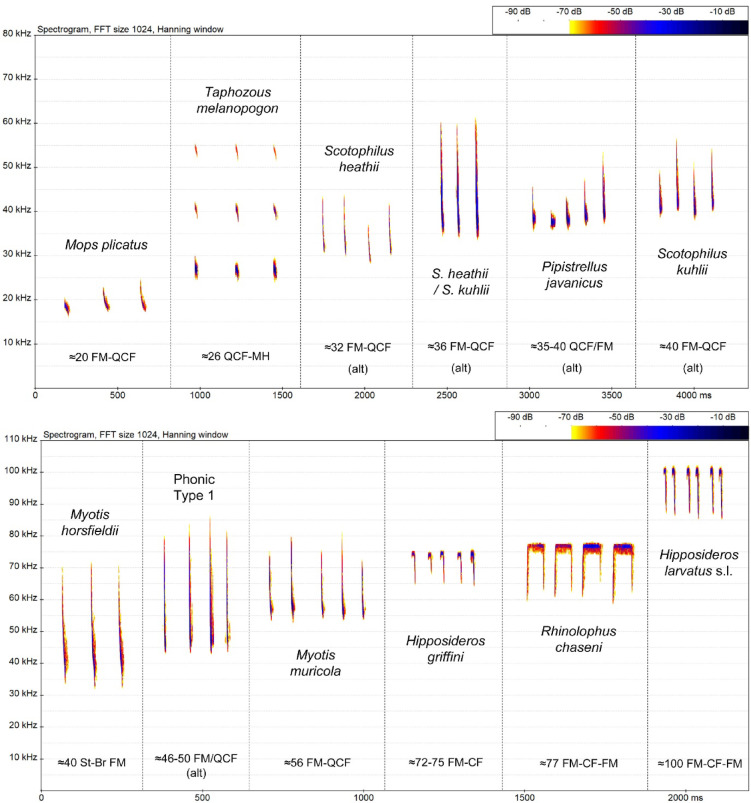
Echolocation call types registered at four wind turbines monitored in study area. Interpulse intervals have been altered to accommodate calls on x-axis. Figures represent lower frequency values (kHz) for each species/call type, followed by abbreviations regarding call variation and structure: alt = alternating calls, CF = constant frequency, FM = frequency-modulated, MH = multi-harmonic, QCF = quasi-constant frequency, StBr = steep broadband.

Twenty-two bat species were recorded during our fieldwork in the study area (Fig. 1; see Supplementary Table S1 & Fig. S1 online). Twelve distinct call types were registered during acoustic monitoring at four wind turbines (WTG), all but one of which (phonic type 1) were identified to species (Fig. 2). Although two were not captured during our live-trapping (*Mops plicatus* and *Taphozous melanopogon*), these emit calls which are readily identified by their characteristic structures and frequencies. Phonic type 1 may represent *Miniopterus magnater*, given its known foraging preferences and local occurrence, coupled with the similarity of the calls registered in monitoring to those emitted by bats captured and released during the fieldwork.

Nine of the twelve call types represented eight species that were particularly common (Table 1). Six of these routinely frequented the four turbines throughout the year, albeit at varying frequencies: *Pipistrellus javanicus* (registered on 97% of nights on average), followed by *Scotophilus kuhlii* (89%), *T. melanopogon* (79%), *S. heathii* / *S. kuhlii* (a call type emitted by both species and therefore not exclusively assignable to either, 76%), *Myotis muricola* (64%) and *M. horsfieldii* (62%). In contrast, the remaining three (*Mops plicatus*,* S. heathii* and phonic type 1) occurred on less than half of the nights monitored on average.

**Table 1:**
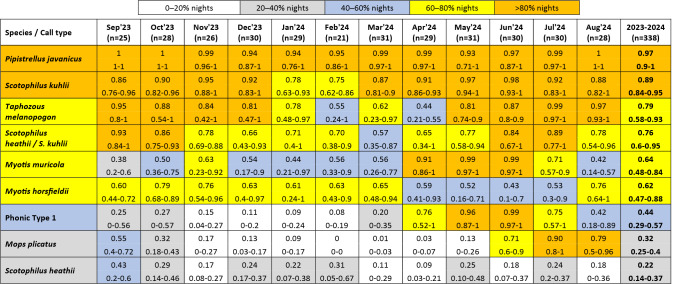
Frequency of occurrence for most common bat species recorded at four wind turbines, September 2023 to August 2024 (*n* = 338 nights). Values given represent the mean, min–max proportion of nights registered across the four turbines monitored.

The remaining three call types registered were far less common and comprised *Hipposideros griffini*, *Rhinolophus chaseni* and *H. larvatus sensu lato* (s.l.). *Hipposideros larvatus* s.l. was registered on just one night at WTG07, whereas *R. chaseni* was registered on 16 nights across the four turbines (four at WTG14, one at WTG20, five at WTG07 & six at WTG10). In contrast, *H. griffini* was registered on 45 nights across the four turbines monitored, although primarily on the eastern side of the wind farm (42 nights = 15 nights at WTG07 & 27 nights at WTG10), with most records there occurring in June (five nights), July (seven), August (five), September (seven) and October (nine) (and ≤ 3 nights in all other months).

### Bat activity at wind turbines

A total of 329,305 bat detections were registered during the study (see Supplementary Table S2 online), giving an average of 6,860 detections per detector-month. While seasonal changes in bat activity were consistent between the four turbines monitored, levels of activity varied greatly with over a third (36.4%, 119,919 detections) registered at WTG20, followed by WTG07 (25.6%, 84,420 detections), WTG14 (23%, 75,866 detections) and WTG10 (14.9%, 49,100 detections).

As measured by mean detections per hour, bat activity was greatest in May–October when this was moderate-to-high (> 13–31 detections/hour) or high (> 31 detections/hour) at WTG14, WTG20 and WTG07, whereas it was greatest (moderate-to-high) at WTG10 in April–July. Relative to these, bat activity was lower in November–April, occurring at moderate levels (> 6–13 detections/hour) at WTG14 and WTG07 and lower again at WTG10, although it remained at moderate-to-high levels at WTG20 for all but one month (January).

**Fig. 3:**
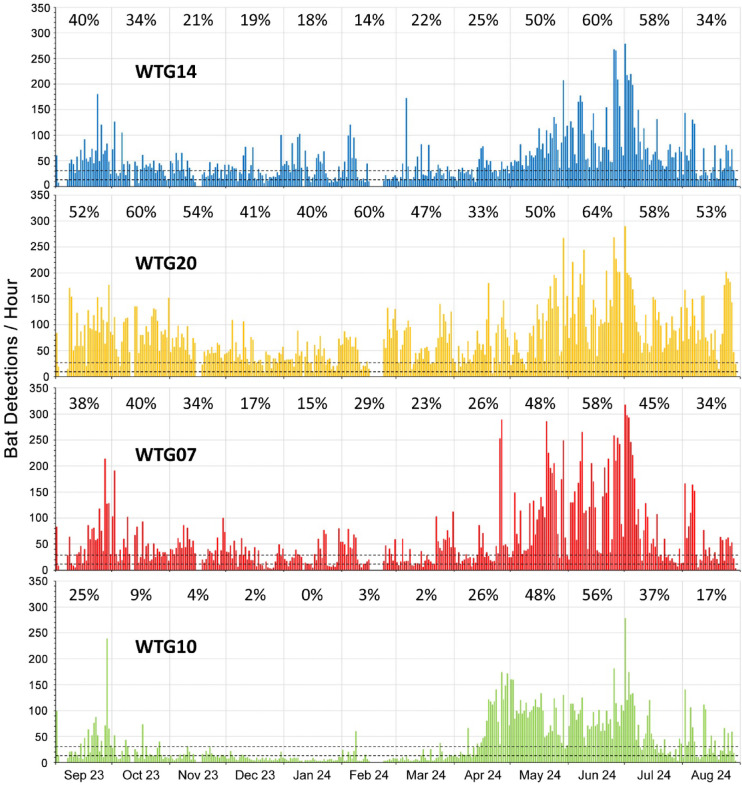
Monthly bat activity at four wind turbines (WTG), September 2023 to August 2024 inclusive. The lower and upper horizontal dashed lines represent the 60th and 80th percentiles (PT) respectively. Figures along the top of each graph represent the combined percentage of night hours monitored each month where bat activity qualified as moderate-to-high (61st–80th PT: >13–31 detections/hour) or high (81st PT: >31 detections/hour).

While helpful as an aggregate summary, monthly averages obscure the fact that bat activity varied considerably on a nightly basis throughout the study and so frequently occurred at moderate-to-high and high levels every month (Fig. 3). Nightly bat activity (= total detections/night) at each turbine was positively correlated between all four turbines monitored over the study period (all values of *p* < 0.001), with pairwise Spearman’s correlation (R_s_) values ranging from 0.512 (WTG20 vs. WTG10) to 0.726 (WTG14 vs. WTG07) and averaging 0.663 (all pairwise combinations).

Nightly patterns of bat activity were similar between the four turbines, although levels of activity between these also varied greatly (see Supplementary Table S3 online). In general, nightly activity was bimodal, being greater between 1800 and 2300 h and 0300–0500 h at three turbines, with a slump between the two periods. The partial exception was WTG10, where activity levels were lower and greatest between 1700 and 1900 h and 0200–0400 h. Based on mean detections per hour, bat activity was moderate-to-high or high from 1800 to 0500 h at three turbines (WTG14, WTG20 & WTG07), whereas levels at WTG10 were moderate-to-high between 1700 and 1900 h and 0200–0400 h and moderate between these times.

**Fig. 4:**
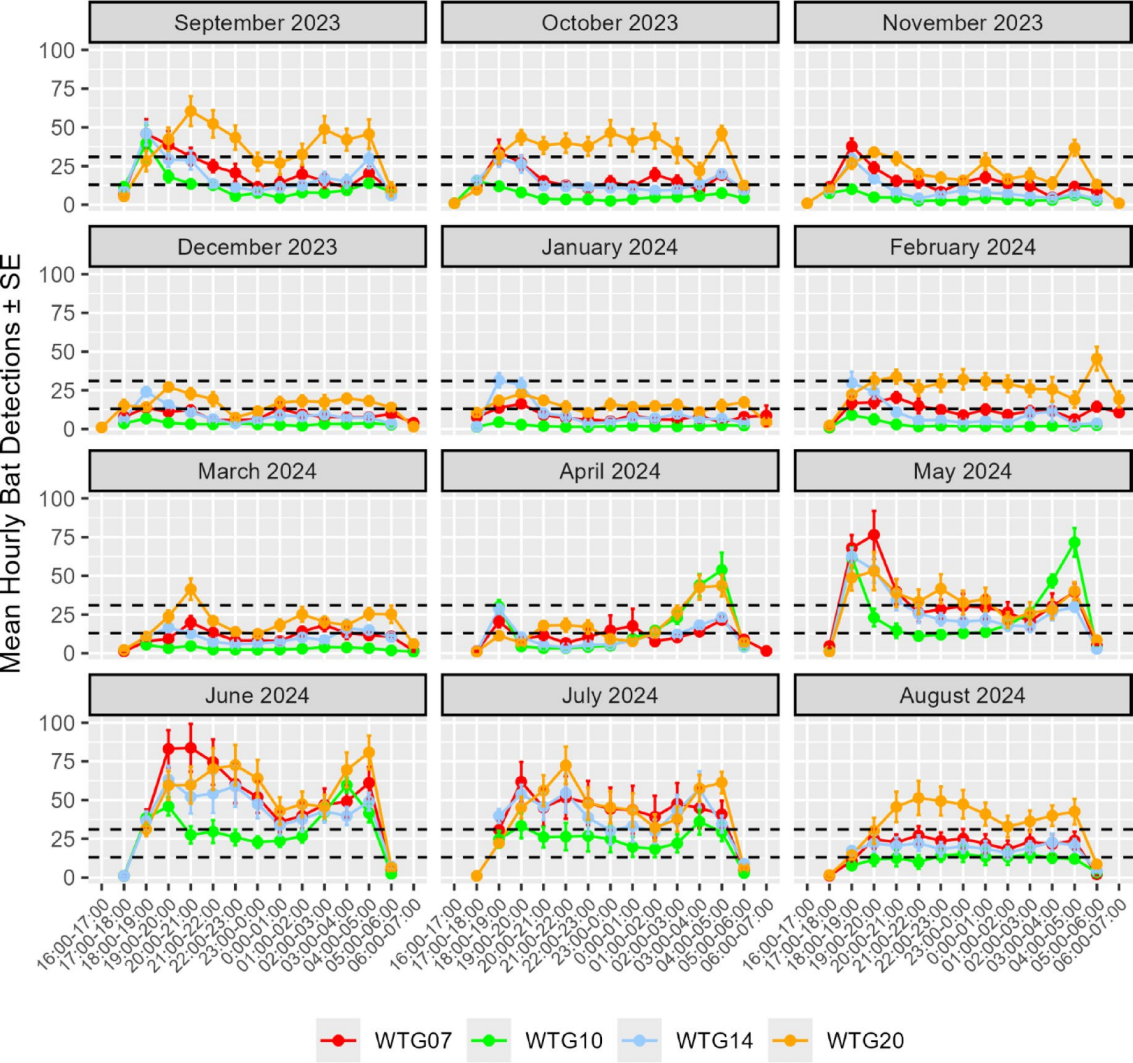
Hourly bat activity at four wind turbines (WTG), September 2023 to August 2024 inclusive. The lower and upper horizontal dashed lines in each graph represent the 60th (13 detections/hour) and 80th percentiles (31 detections/hour), respectively.

As before, monthly averages obscure the fact that nightly activity varied considerably at each location in different months (Fig. 4). For example, while bat activity was continuously above the 50th percentile (9 detections/hour) at WTG20, the post-dusk and pre-dawn peaks and intervening slump in activity noted above were muted or absent in certain months, notably October and December–February. The same was variably true in different months for the remaining turbines (WTG14, WTG07 & WTG10), such that consistent variations between these were not apparent beyond the seasonal highs and lows previously noted.

### Abiotic influences on bat activity

Our hierarchical model yielded a R^2^ value of 0.29, indicating that the use of weakly regularizing priors controlled for underfitting and overfitting of the data, allowing the model to make better predictions. All Rhat values were close to 1.00, indicating adequate chain convergence and reliable parameter estimates, whereas ESS values were also high, similarly indicating good convergence, efficiency and model fit (see Supplementary Table S4 online).

**Fig. 5:**
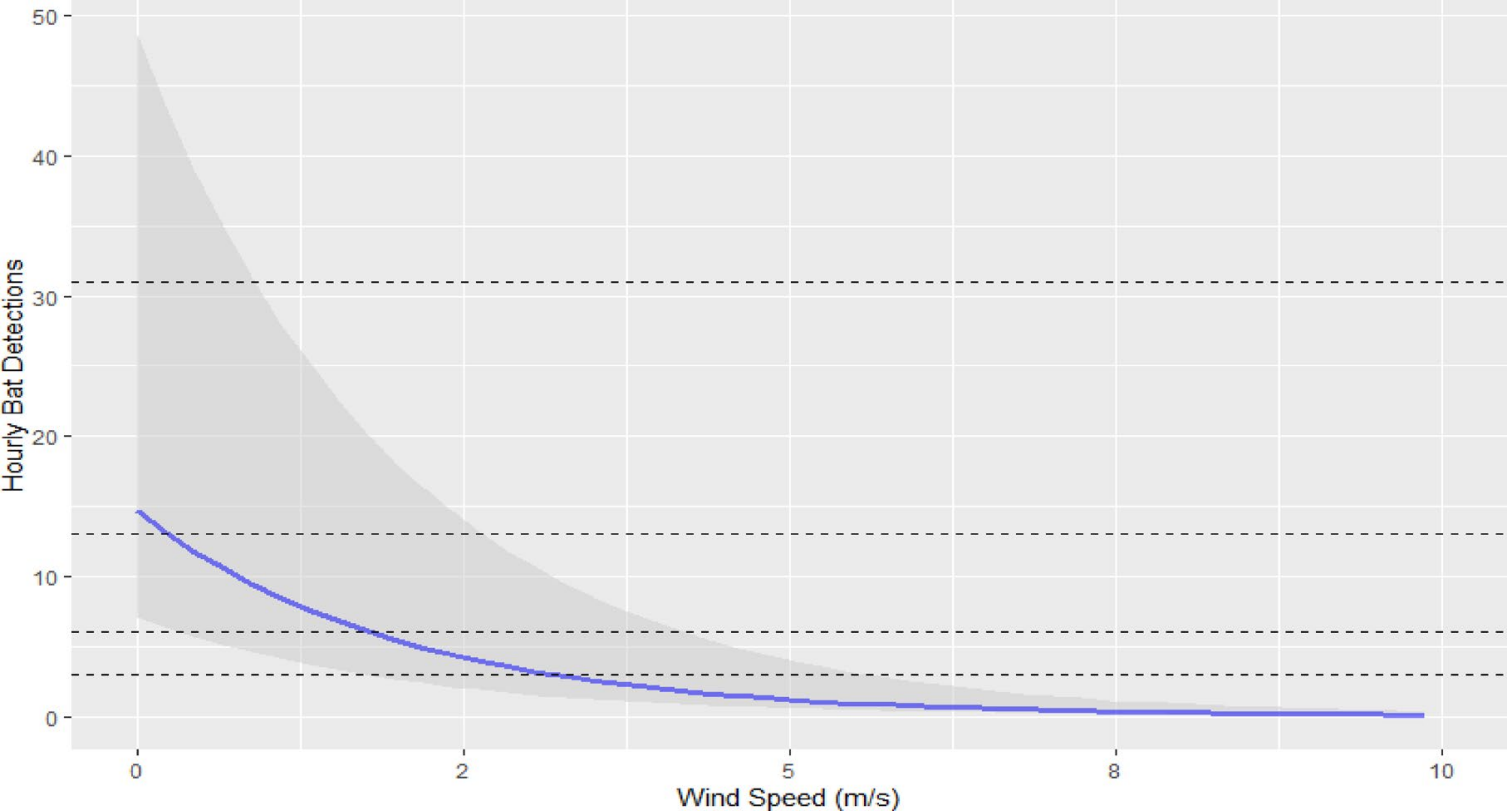
Response of bat activity to wind speed in study area. The blue line represents mean values and the shaded area indicates the 95% confidence intervals. The horizontal dashed lines (lower to upper) indicate the 20th, 40th, 60th & 80th percentiles for bat activity.

**Fig. 6:**
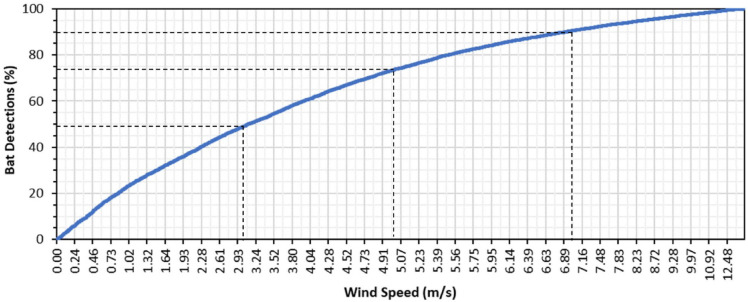
Cumulative proportion of bat detections as a function of windspeeds recorded during the study period (based on 15,184 hourly values).

A strong negative relationship was found between wind speed and bat activity (Est. = -0.50, Est. Error = 0.01). As such, the highest bat activity occurred at low wind speeds, decreasing from a mean values of ≈ 15 bat detections/hour during periods of no wind to less than 1 bat detection/hour once wind speeds exceed 7.0 m/s (Fig. 5). While not directly comparable, this was consistent with the bivariate relationship observed between bat activity and windspeeds during the study, whereby 49%, 74% and 90% of all bat detections during the year occurred at windspeeds of ≤ 3.0 m/s, ≤ 5.0 m/s and ≤ 7.0 m/s, respectively (Fig. 6).

**Fig. 7:**
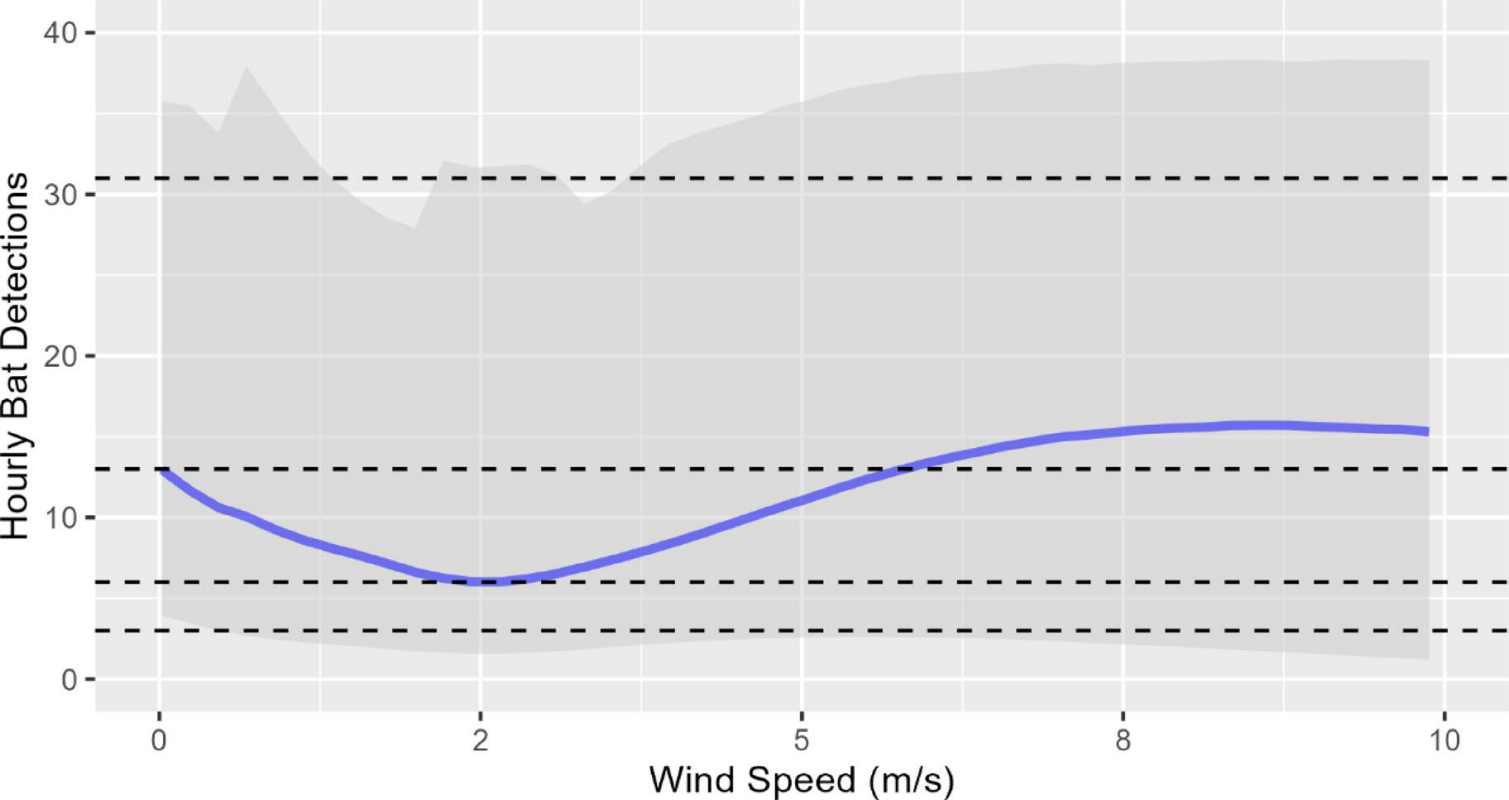
Smooth plots from regression models showing the response of bat activity between 2100–0100 h to wind speeds during dusk and early evening (1700–2100 h). The blue line represents mean values and the shaded area indicates 95% confidence intervals. The horizontal dashed lines (lower to upper) indicate the 20th, 40th, 60th & 80th percentiles for bat activity.

Relative to wind speed, rainfall had an effectively neutral (weakly negative) relationship with bat activity (Est. = -0.01, Est. Error = 0.01), whereas model results indicated a positive relationship between temperature and bat activity (Est. = 0.19, Est. Error = 0.01), such that bat activity increased as temperatures rose and vice versa. When wind speeds during dusk and early evening (1700–2100 h) were < 2 m/s, bat activity in subsequent hours (2100–0100 h) followed the expected decline that typically happens when bats return to night roosts to digest following initial foraging bouts (Fig. 7; see Supplementary Table S5 online). When wind speeds during dusk/early evening exceeded 2 m/s however, rates of subsequent bat activity increased, suggesting a shift in bat activity from the former to the latter. This increase levelled off when wind speeds in dusk/early evening exceeded 8 m/s.

## Discussion

This is the first publicly available study to document patterns of echolocating bat activity and factors influencing these at a windfarm in Southeast Asia. Our findings were somewhat surprising in some regards. One unexpected finding was that our study site supported significant bat activity all year round, while appearing on face value a somewhat harsh environment for bats, being visibly arid, windswept and hypersaline (due to extensive salt farming). While bat activity was consistently greater in May–October, moderate-to-high and high levels of hourly activity often occurred in every month and nightly activity at each location was positively correlated between all locations monitored over the study period. This is consistent with bat activity in northern Vietnam which also peaks in the second and third quarter of each year despite its different climate^25^ and demonstrates the importance of evaluating bat activity on a nightly basis because aggregated measures such as monthly averages obscure important variation. It also supports the notion that some nights have greater bat activity irrespective of location, although there is also potential for spatial shifts. This is because as highly mobile opportunists, insectivorous bats can rapidly respond to sudden and localised increases in prey availability^26,27^.

As expected, bat species registered during the acoustic monitoring formed a subset of the species in the wider area and were mainly taxa adapted to foraging in open space and to a lesser extent, edge/gap environments. Bat species that routinely forage in open spaces have the greatest risk of colliding with turbines^5,28,29^. Three rhinolophoids were also registered on up to 3% of nights, but as the nightly detections mostly comprised single recordings these were presumably commuting rather than foraging bats. Overall, the patterns of occurrence we observed suggest that all but two of the species registered primarily reside in the area throughout the year (*Taphozous melanopogon*, *Pipistrellus javanicus*,* Scotophilus kuhlii*,* S. heathii*,* Myotis muricola* and *M. horsfieldii*). Phonic type 1 and *Mops plicatus* could be partial exceptions, in occurring at moderate to very high frequencies in April–August and June–September (respectively) and very low frequencies outside of these times.

We found rainfall had an effectively neutral relationship with bat activity. While rain can reduce bat activity due to constraints imposed by raindrops on echolocation, increased energetic demands of flight and presumably reduced availability of airborne insect prey^30,31^, our finding is likely due to the fact that the study area naturally experiences little rain and only received roughly half the average yearly total during our sampling (449 mm in 2023–2024 vs. 904–973 mm annually in 1994–2016^32^. As such, rainfall likely has a negligible influence locally in terms of its well-known capacity for reducing bat activity, since the latter is mainly associated with heavy rainfall events. Consequently, rainfall is unlikely to be an important factor for refining blanket curtailment regimes locally to meet the dual requirement of avoiding power losses and bat fatalities.

In contrast, temperature was positively correlated with bat activity. While many studies have shown that bat activity increases with increasing temperature^2^, we suspect that our finding may be partly an artefact of the timing of bat activity, since this typically peaks after dusk when nightly temperatures are also highest, with both declining thereafter. Irrespectively, since the lowest temperatures in the study area rarely fall below 15 °C^32^ and the significance of temperature in reducing bat activity mainly concerns the lower temperatures characteristic of temperate winters (e.g., < 10 °C for prolonged periods^33^ or uplands at higher tropical latitudes (i.e., northern Vietnam at 21–23°N^34^, the lowest temperatures prevailing in the study area would seem unlikely to depress bat activity to a significant extent. As such, temperature is also unlikely to be an important factor for refining blanket curtailment locally.

While rainfall and temperature may have limited utility for refining blanket curtailment in the study area, this is unlikely to be true of many areas with higher rainfall and/or distinct winters throughout the region. For example, the former includes most of the Southeast Asian mainland where a wet season with significantly greater rainfall prevails from May to October^35^, the months of greatest bat activity annually^25^. The latter includes higher elevations in mountainous areas and latitudes above 16°N in Vietnam, where temperatures drop significantly in December–February^34^. Both are also true of many areas designated for development of wind energy in Lao PDR. These include Xekong Province (15° N) in southern Lao PDR where the largest wind farm in Southeast Asia recently began operations^9^. As such, we advocate for site-specific studies to quantify the utility of these factors for modifying curtailment regimes to maximise power production while minimising bat fatalities.

Not surprisingly, our results were consistent with the well-known relationship between bat activity and windspeed. Since this relationship reflects the decreasing potential for bat fatalities with increasing windspeeds, with 74% of all bat detections occurring at windspeeds of ≤ 5.0 m/s, it has a direct bearing on potential curtailment options. Further, while bat activity typically peaks in the immediate post-dusk and pre-dawn hours and declines between these, our data indicate this pattern is also influenced by wind speeds. When post-dusk wind speeds are low (< 2 m/s), the decrease in subsequent activity reflects the typical decrease in bat activity as the night progresses, whereas when post-dusk wind speeds are greater (> 2 m/s), bats shift their activity to later hours when foraging is less costly. This is consistent with optimal foraging theory, whereby an individual would choose not to forage until wind speeds are at a level that allow this to yield a net energetic benefit. While there is a large amount of uncertainty in our estimates, this pattern is clear and also has implications for refining curtailment regimes in the region.

Notwithstanding these findings, our results also indicate that over 70% of the variance observed in bat activity was driven by variables not included in our hierarchical model. While we can presently only speculate as to what these factors may be, future studies could consider incorporating variables such as proximity to roosts and water sources, differences in habitats in adjacent areas and tracking variations in insect prey availability. More broadly, our findings regarding the effect of windspeed on bat activity are consistent with studies worldwide^2,3,20^ and likewise suggest that curtailment at low wind speeds will be an effective strategy for reducing bat fatalities at windfarms in Southeast Asia. Much remains to be learnt however, not least regarding the potential of multicriteria algorithm-based and context-dependent curtailment methods^3,23^ for maximising energy production while avoiding bat fatalities. Given the deficit of publicly-available information on bat fatalities at wind farms in Southeast Asia and the region’s undeniable importance for global bat diversity, we support recent calls for government and lending institutions to insist on conservation standards as a condition of financing or subsidizing wind energy projects^3^. For large projects situated in natural areas particularly, these should include post-construction monitoring and release of the results to the public, although this would by no means be necessary for small projects in areas that are demonstrably of low risk.

In conclusion, we consider that significant reductions in bat mortality due to collisions with turbines can likely be achieved by tailored curtailment protocols, with more limited impacts on power production than blanket curtailment. Because local context is very important however, protocols for tailored curtailment need to be designed on the basis of site-specific information provided by monitoring of bat activity during turbine operations (ideally including at nacelle height, not just lower down). To this end, we reiterate that the lack of a strong relationship between rainfall and bat activity at our study site is unlikely to be the case in the more humid, monsoon-driven regions of Vietnam and broader Southeast Asia. Monitoring of fatalities is also crucial to understanding the effectiveness of curtailment programmes and adaptively managing these, whereas public release of the results of bat activity and fatality monitoring is similarly essential given the major gaps in existing knowledge.

## Methods

### Study site

Our sampling was undertaken at the ACEN wind farm which comprises 22 wind turbines (GE Cypress 4-158) with a total capacity of 88 MW in the coastal area of Ninh Thuan Province, southern Vietnam (Thuan Nam district: 11.399261°N, 108.892862°E). The wind farm occupies 30.77 ha and is situated along national highway 1A, which traverses the site on a north-south axis close to the southern border with Binh Thuan Province (Fig. 1a). The 22 turbines have a hub height of 121 m, a rotor diameter of 158 m (giving a blade-swept area of 19,607 m^2^, 42–200 m above ground) and a design cut-in speed of 3 m/s^32^. The site began operations in September 2021, although 5 m/s has been applied as a cut-in speed during night hours under a blanket curtailment regime since March 2023^36^.

The topography of the wind farm is flat and open (below 50 m elevation), although its southern and central portions are bounded by rocky mountains to the west/southwest and east/southeast, such that the site partly occupies the mouth of a north-south valley separating these (Fig. 1a). Habitats in the area mostly comprise several modified types including large areas of arid scrubland, agricultural vegetation (planted trees and annual crops), modified seasonal wetlands, waterbodies (streams, ponds) and extensive areas of salt ponds (Fig. 1b), solar parks and other wind farms. Habitats adjacent to the wind farm include the rocky mountains (which reach ≈ 850 m in elevation) and rice fields and tree plantations in lowland areas to the immediate north. No caves are known in the region.

The area has a tropical monsoon climate with autumn-winter rains^34^. The wet season typically runs from September to December and the dry season from January to August, with coastal areas experiencing the lowest rainfall. Long term data from the closest meteorological stations (Quan Te & Ca Na, both within 5 km^32^ indicate that the driest period occurs in January–April (< 30 mm /month), after which rainfall increases in May–August (56–108 mm /month) and peaks in September–November (134–212 mm /month), with December (73–98 mm) being transitional between the two main seasons. The prevailing winds come from the NNE in October to March and from the S-SSW in April to September and are strongest in December–February (averaging 7.8–8.8 m/s) and June–August (7.6–8.7 m/s)^32^. Average temperatures vary little, ranging from 24.8 °C in January to 28.9 °C in May each year^32^.

Hourly temperature and wind speeds during the study were registered by recording units onboard all of the turbines monitored, whereas hourly rainfall data were obtained from a weather station centrally located (11.438362°N, 108.873776°E) in the study area. Rainfall totalled only 449 mm during the study period. No rain was recorded in January–April 2024, whereas 290 mm fell in September–December 2023 and 159 mm in May–August 2024. Prevailing winds were from NNE–NE in November–March and S–SSW in April–October. On average, winds were strongest in November–February (with monthly means of 6.1–9.4 m/s) and July–August (6.2 m/s in either month) and notably weaker in other months e.g., 5.0–5.7 m/s in March–June and 3.8–5.2 m/s in September–October. Mean monthly temperatures varied from 25.5 to 27.1 °C at individual turbines in January (with an absolute hourly low of 21.3 °C in December 2023) to 30.4–32.0 °C in May.

### Live-trapping and call sampling for bats

While only six bat species were identified in environmental assessments undertaken in 2019–2020^32^, our desk review indicated as many as 33 bat species could occur locally, while predicting the total number present as somewhat less^37^. As such, live trapping was undertaken at the site to confirm the bat species present and obtain reference recordings of echolocation calls to enable their acoustic identification. All study protocols and procedures were approved by local authorities and the Research Committee of the Institute of Biology (formerly the Institute of Ecology and Biological Resources), Vietnam Academy of Science and Technology (Dispatch No. 624/STTNSV, 14 May 2023). Methods employed for live-trapping, handling and processing of bats conformed with the guidelines of Sikes and the Animal Care and Use Committee of the American Society of Mammalogists^38^. The study is reported in accordance with ARRIVE guidelines (https://arriveguidelines.org).

Due to the flat and open terrain and habitats of the site, trapping effort necessarily focused on freshwater ponds (Fig. 1c), day roosts in urban areas and putative flyways along hedgerows, streams and other linear breaks in scrub vegetation in the adjacent foothills. Bats were live-trapped using four-bank harp traps, mobile traps and 70-denier mist nets of various sizes (e.g., 7 × 3 m, 10 × 3 m, 12 × 3 m) for 56 trap nights between July 2023 and February 2024 (typically eight trap nights per month). Live-sampling was avoided on consecutive nights at the same locations to avoid trap familiarity. All bats captured were measured, photographed and identified in the field based on appropriate field guides^39,40^ and the first two authors taxonomic experience of Indochinese bats (> 45 years combined).

Sampling led to the live-capture of 630 bats representing 21 species belonging to seven families, plus one carcass of *Taphozous melanopogon* which was found underneath a wind turbine in 2023. The bats caught included three frugivorous species (Pteropodidae, 109 bats) which were omitted from analysis because they do not use laryngeal echolocation (although some species employ a rudimentary form of biosonar based on atonal clicks^39,40^. All but one of the 22 species are considered Least Concern^41^, the exception being *Hipposideros griffini* which is regarded as Near-Threatened^42^. They also include eight taxa which were predicted to have a high risk of bat-turbine collisions due to their foraging behaviour. This is supported by empirical data, as seven of these species have been recorded in fatality monitoring efforts at the site and nearby wind farms^36^(see Supplementary Table S1 online).

Reference calls were recorded from all echolocating bats captured to enable identification of unseen bats registered in the passive acoustic monitoring. A M500-384 USB ultrasound microphone (Pettersson Electronik AB, Sweden), connected to an android smartphone (Samsung Galaxy S6) running the Bat Recorder app (version 1.0R156) was employed to this end. As the focus was on sampling calls emitted by flying bats, a flight tent (= 6 × 2 × 2 m) was used to record the calls of smaller-bodied taxa (particularly smaller rhinolophids and hipposiderids with low amplitude calls) in flight, whereas a larger tent (= 10 × 5 × 3.5 m), enclosure (= 17 × 9 × 4.5 m) and hand-releases in open areas were employed for megadermatids, vespertilionids, miniopterids and larger rhinolophids and hipposiderids.

### Passive acoustic monitoring

Following a one-month trial with four devices in August 2023 (= 116 detector-nights), Song Meter 4 full spectrum bat detectors with SMM-U2 microphones (Wildlife Acoustics, USA) were installed at the base of four wind turbines (WTG14, WTG20, WTG07 & WTG10: Fig. 1a). These were chosen to maximise physical coverage of the wind farm and were visited for maintenance and data retrieval each month from 1 September 2023 to 29 August 2024. This resulted in 338 nights of sampling per detector and a study total of 1,352 detector-nights.

The sampling range of bat detectors is limited and frequency-dependent, typically reaching up to 30 m for *Pipistrellus* spp. calling at ≈ 45 kHz, although sometimes further depending on the orientation of the bat, weather conditions and call volume^19^. Because our detectors were deployed at the base of turbines and not within the blade-swept areas (42–200 m above ground), it was recognized that these would mainly register bats echolocating at lower-frequencies within the lower portion of the blade-swept zones and areas below this, as well as higher-frequency echolocators in the latter. However, it was also recognised that all bats that regularly fly in the open spaces around wind turbines routinely risk collisions with the turbine blades due to their exceptional vertical mobility.

All microphones were positioned ≈ 3 m above ground level, orientated 90° from vertical (i.e., horizontal to the ground) and tested to confirm adequate sensitivity before and after deployment. On all nights, the detectors were programmed to record from 30 min before sunset to 30 min after sunrise with the following settings: gain = 12 dB, 16k high filter = on, sample rate = 384 kHz, minimum duration = 1.5 ms, minimum trigger frequency = 16 kHz, trigger level = 12 dB, trigger window = 1 s, maximum length = 10 s.

### Acoustic species identification

To identify unseen bats registered in the acoustic monitoring, we created a reference library of species echolocation calls based on flight recordings obtained from bat species identified during the live-trapping. Following vetting of individual recordings, the validated reference data comprised 733 recordings of 508 bats representing 18 species (see Supplementary Table S6 online). The recordings and the associated metadata can be accessed at www.chirovox.com^43^, via accession numbers A005100–A005832.

Following this, recordings of sequences of distinct call types produced by free-flying bats at the site were collated through manual examination of the 16,116 anonymous recordings generated during the one-month trial. The various call types present were identified based on the validated reference data combined with verified recordings for species from elsewhere in Vietnam and Cambodia^44–46^. Call parameters were extracted from the recordings for each species and call type using SCAN’R software (Binary Acoustic Technology, USA).

These data were employed in a discriminant function analysis to determine the occurrence of species and call types at each monitoring location on a nightly basis using a filtering pipeline in R software (R Core Team, Austria) and SZAPP software^47,48^. This method comprises a semi-automated procedure for filtering and inspecting massive datasets which provides complete transparency and control over the analysis. This notably allows the rate of false positive identifications to be reduced to zero, whereas the potential for false negative detections is dependent on the feature extraction process and diligence of the analyst^49^.

### Quantifying bat activity

Following Kunz et al. (2007)^4^, we defined a bat detection as a sequence of > 2 echolocation calls, with each sequence (or detection) separated by > 1 s. The second criterion was achieved by employing a 1 s trigger window on all of the detectors, whereas the first was achieved in subsequent processing. Due to the vast number of recordings generated by the four detectors (> 1,275,000 files) over the study period, we created a filter in SCAN’R to identify and remove recordings not containing at least 3 bat signals (first criterion). This ensured that all recordings ‘passed’ by the filter met our definition for a bat detection.

The performance of the filter was evaluated by manually verifying all recordings ‘passed’ by the filter which were generated during the one-month trial in August 2023 (*n* = 16,116 out of 138,780 files). This process indicated the filter was effective, with 97% of the passed recordings (15,675 files) containing bat signals. While it also means the number of detections were artificially inflated by 3%, this was compensated for by the fact that some recordings contained signals from more than one bat, such that on balance, our dataset represents a conservative sample. This was regarded as sufficient for subsequent analysis, partly as the alternative would be to manually examine the entire post-filter dataset (329,305 files). In addition to being enormously time-consuming, this was regarded as unnecessary because it would not alter the primary signals of the data.

Percentiles were employed to ensure consistency in comparisons of activity between different areas and times. The categories of Lintott et al. (2017)^50^ and Richardson et al. (2021)^19^ were adopted to this end because these have been widely employed in assessments of bat activity at wind farms and other sites, as follows: (1) low activity = 0–20th percentiles (≤ 3 detections/hour), (2) low-to-moderate activity = 21st–40th percentiles (> 3–6 detections/hour), (3) moderate activity = 41st–60th percentiles (> 6–13 detections/hour), (4) moderate-to-high activity = 61st–80th percentiles (> 13–31 detections/hour), (5) high activity = 81st–100th percentiles (> 31 detections/hour).

### Modelling influences on bat activity

A two-level Bayesian hierarchical model was fitted to evaluate the relationship between hourly bat activity and three explanatory variables (rainfall, temperature and wind speed), whereby the two hierarchical levels were the locations monitored and month of monitoring. To evaluate the effect of wind speeds during dusk and early evening on subsequent bat activity, we used wind speeds during the first four hours of each session (1700–2100 h) to predict hourly bat detections in the following four hours (2100–0100 h). A generalized additive model was employed to this end, as these allow for non-linear relationships.

Before inclusion in the models, all continuous predictors were standardized to a mean of 0 and a standard deviation of 1 to improve model convergence and comparability of effect sizes. The models were fitted with a negative binomial distribution (a distribution with a high maximum entropy for a response variable of counts), which fit the data better than a Poisson distribution. Priors were chosen to be weakly regularizing so as to control for both under and overfitting of the model to the data. The models were fitted with four chains, each with 2,000 iterations, with 500 warmup iterations. Convergence criteria such as effective sample sizes and R-hat values were used to check for appropriate model convergence throughout and trace plots were inspected for signs of incomplete mixing when necessary. Non-centred parameterization was employed to facilitate convergence and efficient sampling of chains. R^2^ values (defined as the variance of the predicted values divided by the variance of the predicted values plus the variance of the errors) were employed to assess how much variation in the data was explained by the model. Higher R^2^ values indicate that more variation in the data is explained by a model, although high values can sometimes result in poor predictions.

All models were fit using the ‘*brms*’ package^51^ and model results and visualizations were generated using the ‘*tidybayes-rethinking*’ and ‘*ggplot2*’ packages^52,53^ in R studio^54^.

## Supplementary Information

Below is the link to the electronic supplementary material.

Supplementary Material 1

## Data Availability

The data and R code employed for processing, visualisation and analysis in this study are available from https://github.com/bhopalstiffs/VietnamBats_Windfarms.
