# Supplementary material for: Establishing baselines for echolocating bat activity at wind farms in mainland Southeast Asia
Source: Sci Rep. 2026 Feb 27;16:10207. doi: 10.1038/s41598-026-41384-8 (PMC13022042; doi:10.1038/s41598-026-41384-8)
Supplement: Supplementary file 1 — Supplementary Material 1 [file 41598_2026_41384_MOESM1_ESM.pdf]

Establishing baselines for echolocating bat activity at wind farms in mainland Southeast Asia

Neil M. Furey[1,*], Vuong Tan Tu[2], Alan Hitch[3,4], John Pilgrim[5] & Mark Kunzer[6]

[1] Harrison Institute, 15 St. Botolphs Road, Sevenoaks TN13 3AQ, United Kingdom. * Email: neil.m.furey@gmail.com

[2] Institute of Biology, Vietnam Academy of Science and Technology, No. 18, Hoang Quoc Viet Road, Nghia Do, Hanoi, Vietnam.

[3] Museum of Wildlife and Fish Biology, Department of Wildlife, Fish, and Conservation Biology, University of California at Davis, Davis, CA 95616, USA.

[4] School of Data Analytics and Computational Sciences, Harrisburg University of Science and Technology, Harrisburg, PA 17101, USA.

[5] John Pilgrim Limited, The Old Post Office, The Street, Boyton, Woodbridge, Suffolk, UK, IP123LW.

[6] (retired) Private Sector Investment Funds and Special Initiatives Division, Private Sector Operations Department, Asian Development Bank, 6 ADB Avenue, Mandaluyong, Metro Manila, Philippines.

Supplementary Table S1

Bat species recorded during fieldwork in study area

No.	Family / Species	No of individuals	IUCN Status	Population Trend	Foraging Strategy	Predicted Collision Risk
**I**	**Pteropodidae**					
1	*Cynopterus sphinx*	3	LC	Increasing	IV	Medium
2	*Eonycteris spelaea*	38	LC	Decreasing	V	High [C]
3	*Rousettus* spp. [A]	68	-	-	V	High [C]
**II**	**Emballonuridae**					
4	*Taphozous melanopogon* [B]	0	LC	Stable	III	High [C]
**III**	**Megadermatidae**					
5	*Lyroderma lyra*	2	LC	Unknown	II	Medium
6	*Megaderma spasma*	17	LC	Unknown	II	Medium
**IV**	**Rhinolophidae**					
7	*Rhinolophus affinis*	41	LC	Stable	I	Medium
8	*Rhinolophus chaseni*	15	NE	-	I	Low
9	*Rhinolophus pearsonii*	16	LC	Unknown	II	Medium
10	*Rhinolophus pusillus*	68	LC	Stable	I	Low
**V**	**Hipposideridae**					
11	*Hipposideros cineraceus*	12	LC	Unknown	I	Low
12	*Hipposideros galeritus*	18	LC	Unknown	I	Low
13	*Hipposideros gentilis*	68	LC	Unknown	I	Low
14	*Hipposideros griffini*	17	NT	Decreasing	II	Medium
15	*Hipposideros larvatus* s.l.	49	LC	Unknown	II	Medium
**VI**	**Vespertilionidae**					
16	*Myotis horsfieldii*	38	LC	Stable	II	Medium
17	*Myotis muricola*	1	LC	Stable	II	Medium [C]
18	*Pipistrellus javanicus*	55	LC	Stable	III	High [C]
19	*Scotophilus heathii*	54	LC	Stable	III	High [C]
20	*Scotophilus kuhlii*	42	LC	Stable	III	High [C]
**VII**	**Miniopteridae**					
21	*Miniopterus magnater*	4	LC	Unknown	III	High
22	*Miniopterus pusillus*	4	LC	Unknown	III	High
		**630**				

IUCN (2024) Status: LC=Least Concern, NE=Not evaluated, NT=Near Threatened. Population Trend per IUCN (2024).

Foraging Strategies (per McKenzie et al. 1995): [I] Insectivorous species that forage in the highly cluttered airspace within the forest interior (forest interior specialists); [II] Insectivorous species that forage in partially cluttered spaces such as clearings, streams or other tunnels within the forest or just above the canopy (edge and gap foragers); [III] Insectivorous bats that forage in unobstructed airspaces found in large clearings or high above the forest canopy (open-space foragers); [IV] Fruit and nectar-eating bats that fly into the partially cluttered air-spaces between tree canopies, roost in small numbers and forage locally; [V] Fruit and nectar-eating bats that fly in unobstructed airspaces, roost in large colonies and forage over large areas. Species assigned per Kruskop (2013) and Furey & Racey (2016).

Predicted Collision Risk: In decreasing order, the risk of collision with turbines typically associated with the above foraging strategies is Strategy III > Strategy V > Strategy IV > Strategy II > Strategy I. These categories translate into the following risk predictions: High = strategy III and V species, Medium = strategy IV and II species, Low = strategy I species. Although published data are very scarce for SE Asia, these predictions are supported by unpublished data from fatality monitoring efforts at windfarms in the Ninh Thuan and Thuan Binh provinces.

[A] No attempt was made to identify *Rousettus* bats to species (although these represent either *R. leschenaultii* [Near-Threatened: IUCN 2024] or *R. amplexicaudatus* [Least Concern: IUCN 2024]) because these can only be reliably distinguished by the shape of the lower rear molars (elongated in *leschenaultii*, subcircular in *amplexicaudatus*) whose confirmation typically requires euthanasia.

[B] Comprising a single carcass found beneath a wind turbine in 2023.

[C] Recorded in fatality monitoring efforts, either at the study site (SIE 2024) or nearby wind farms.

References

Furey, N.M., Racey, P.A. (2016) Can wing morphology inform conservation priorities for Southeast Asian cave bats? Biotropica 48: 545–556.

Kruskop, S.V. (2013) Bats of Vietnam, checklist and an identification manual. Joint Russian Vietnamese Science and Technological Tropical Centre, Hanoi, Vietnam.

McKenzie, N.L., Gunnell, A.C., Yani, M., Williams, M.R. (1995) Correspondence between flight morphology and foraging ecology in some palaeotropical bats. Australian Journal of Zoology 43: 241–457.

[SIE] Southern Institute of Ecology (2024) Biodiversity monitoring at BIM wind power plant in Ninh Thuan Province. Post construction bird and fatality monitoring from October 2023 to October 2024. Consultancy report to BIM Wind Power JSC.

Supplementary Figure S1

Indicative images of bat species recorded during fieldwork
(all images are of bats encountered in the study area)

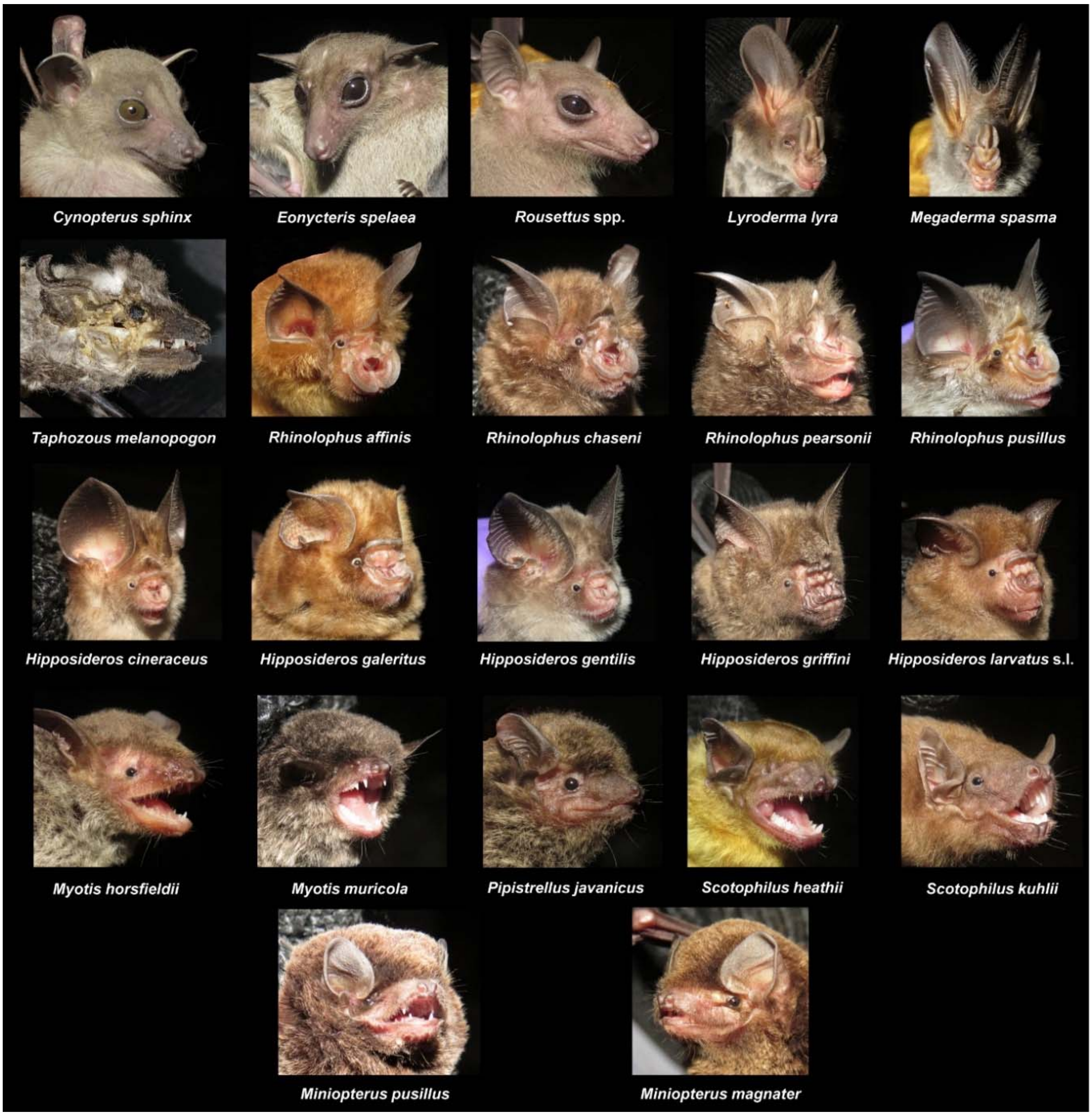

Supplementary Table S2

Monthly bat activity at wind turbines (WTG), September 2023 to August 2024 inclusive. Values represent total detections / mean detections per hour (d/hr).

Activity (d/hr)	Low (≤3)	Low-Moderate (>3–6)	Moderate (>6–13)	Moderate-High (>13–31)	High (>31)

Month	WTG14	WTG20	WTG07	WTG10	Overall
September '23	5,580 / 17.2	10,607 / 28.3	5,907 / 16.9	3,678 / 10.5	2,5772 / 18.4
October '23	4,999 / 13.7	11,480 / 27.3	5,417 / 13.8	1,944 / 5.0	23,840 / 15.2
November '23	2,989 / 8.8	6,962 / 17.9	4,729 / 13.0	1,390 / 3.8	16,070 / 11.0
December '23	3,222 / 8.3	5,903 / 13.1	2,695 / 6.4	1,061 / 2.5	12,881 / 7.7
January '24	3,483 / 9.2	5,360 / 12.3	2,638 / 6.5	544 / 1.3	12,025 / 7.4
February '24	2,255 / 8.3	7,586 / 24.6	3,008 / 10.2	557 / 1.9	13,406 / 11.5
March '24	3,827 / 9.5	7,472 / 17.2	3,989 / 9.2	1,001 / 2.3	16,289 / 9.6
April '24	3,870 / 10.3	5,875 / 14.5	4,100 / 10.1	6,029 / 14.8	19,874 / 12.5
May '24	10,163 / 25.2	12,257 / 28.2	12,814 / 29.5	9,516 / 21.9	44,750 / 26.2
June '24	15,616 / 40.0	19,098 / 45.5	18,114 / 43.1	11,450 / 27.3	64,278 / 39.0
July '24	13,816 / 35.4	15,380 / 36.6	14,476 / 34.5	8,291 / 19.7	51,963 / 31.5
August '24	6,046 / 16.6	11,939 / 30.5	6,533 / 16.7	3,639 / 9.3	28,157 / 18.6
Total	75,866	119,919	84,420	49,100	329,305

Supplementary Table S3

Nightly bat activity at wind turbines (WTG), September 2023 to August 2024 inclusive. Values represent total detections / mean detections per hour (mean d/hr).

Activity (mean d/hr)	Low (≤3)	Low-Moderate (>3–6)	Moderate (>6–13)	Moderate-High (>13–31)	High (>31)

Hours	WTG14	WTG20	WTG07	WTG10	Overall
16:00–17:00	0	3 / 0.0	0	15 / 0.0	18 / 0.0
17:00–18:00	998 / 3.0	1,285 / 3.8	1,235 / 3.7	6,052 / 17.9	9,570 / 7.1
18:00–19:00	10,788 / 31.9	7,802 / 23.1	9,389 / 27.8	5,589 / 16.5	33,568 / 24.8
19:00–20:00	10,222 / 30.2	11,559 / 34.2	11,259 / 33.3	3,630 / 10.7	36,670 / 27.1
20:00–21:00	7,079 / 20.9	12,714 / 37.6	8,982 / 26.6	3,149 / 9.3	31,924 / 23.6
21:00–22:00	6,103 / 18.1	11,968 / 35.4	7,423 / 22.0	2,981 / 8.8	28,475 / 21.1
22:00–23:00	5,440 / 16.1	10,313 / 30.5	6,476 / 19.2	2,664 / 7.9	24,893 / 18.4
23:00–00:00	4,908 / 14.5	9,365 / 27.7	6,187 / 18.3	2,737 / 8.1	23,197 / 17.2
00:00–01:00	4,769 / 14.1	9,032 / 26.7	5,926 / 17.5	3,054 / 9.0	22,781 / 16.8
01:00–02:00	4715 / 13.9	8,574 / 25.4	5,643 / 16.7	4,222 / 12.5	23,154 / 17.1
02:00–03:00	5,615 / 16.6	9,020 / 26.7	5,802 / 17.2	6,413 / 19.0	26,850 / 19.9
03:00–04:00	6,549 / 19.4	10,548 / 31.2	6,120 / 18.1	6,789 / 20.1	30,006 / 22.2
04:00–05:00	6,890 / 20.4	13,102 / 38.8	7,445 / 22.0	1,290 / 3.8	28,727 / 21.2
05:00–06:00	1,790 / 15.3	4,188 / 12.4	2,296 / 6.8	515 / 1.5	8,789 / 6.5
06:00–07:00	0	446 / 1.3	237 / 0.7	0	683 / 1.0

Supplementary Table S4

Hierarchical Bayesian model examining the influence of wind speed, rain and temperature on bat activity. ESS = effective sample sizes.

Multilevel Hyperparameters							
~ Location (Number of levels: 4)							
	Estimate	Est. Error	l-95% CI	u-95% CI	Rhat	Bulk ESS	Tail ESS
sd (Intercept)	0.63	0.44	0.22	1.84	1.00	1426	1686
~ Month (Number of levels: 12)							
sd (Intercept)	0.47	0.12	0.30	0.77	1.01	923	2100
Regression Coefficients							
Intercept	2.75	0.39	1.94	3.50	1.00	1577	1583
Wind speed	-0.50	0.01	-0.52	-0.48	1.00	6310	3882
Rainfall	-0.01	0.01	-0.02	0.01	1.00	6207	3565
Temperature	0.19	0.01	0.17	0.20	1.00	6101	3789

Supplementary Table S5

Generalized additive hierarchical Bayesian model examining the influence of wind speeds during dusk and early evening (1700–2100 hrs) to subsequent bat activity (2100–0100 hrs). ESS = effective sample sizes.

Multilevel Hyperparameters							
~ Location (Number of levels: 4)							
	Estimate	Est. Error	l-95% CI	u-95% CI	Rhat	Bulk ESS	Tail ESS
sd (Intercept)	1.01	0.61	0.40	2.71	1.00	571	503
~ Month (Number of levels: 12)							
sd (Intercept)	0.67	0.16	0.44	1.05	1.01	489	726
Regression Coefficients							
Intercept	2.54	0.60	1.27	3.69	1.00	532	501
Wind speed	-1.53	1.29	-4.03	1.06	1.00	1032	857

Supplementary Table S6

Validated call recordings obtained for bat species in study area

All of the recordings below and their metadata can be accessed without restriction at www.chirovox.com (Gorfol et al. 2022) via accession numbers A005100–A005832

No	Family / Species	No of Captures	Reference Recordings [C]		
			Resting	Enclosure	Hand Release
**I**	**Pteropodidae**				
1	*Cynopterus sphinx*	3	-	-	-
2	*Eonycteris spelaea*	38	-	-	-
3	*Rousettus* spp.	68 [A]	-	-	-
**II**	**Emballonuridae**				
4	*Taphozous melanopogon*	0 [B]	-	-	-
**III**	**Megadermatidae**				
5	*Lyroderma lyra*	2		2	
6	*Megaderma spasma*	17		16	2
**IV**	**Rhinolophidae**				
7	*Rhinolophus affinis*	41	23	35	9
8	*Rhinolophus chaseni*	15	4	13	4
9	*Rhinolophus pearsonii*	16	4	14	1
10	*Rhinolophus pusillus*	68	3	62	8
**V**	**Hipposideridae**				
11	*Hipposideros cineraceus*	12	3	8	
12	*Hipposideros galeritus*	18	5	17	3
13	*Hipposideros gentilis*	68	18	65	2
14	*Hipposideros griffini*	17	5	3	13
15	*Hipposideros larvatus* s.l.	49	10	47	
**VI**	**Vespertilionidae**				
16	*Myotis horsfieldii*	38		37	25
17	*Myotis muricola*	1		1	
18	*Pipistrellus javanicus*	55		48	44
19	*Scotophilus heathii*	54		45	44
20	*Scotophilus kuhlii*	42		39	37
**VII**	**Miniopteridae**				
21	*Miniopterus magnater*	4		4	3
22	*Miniopterus pusillus*	4		4	3
		**630**	**75**	**460**	**198**

[A] No attempt was made to identify *Rousettus* bats to species (although these represent either *R. leschenaultii* [NT] or *R. amplexicaudatus* [LC]) because these can only be reliably distinguished by the shape of the lower rear molars (elongated in *leschenaultii*, subcircular in *amplexicaudatus*) whose confirmation typically requires euthanasia.

[B] Comprising a single carcass found beneath a wind turbine in 2023.

[C] Figures represent numbers of bats sampled for each recording scenario, with more than one scenario often recorded from an individual bat.
